# The right journal for the right time - *Cell & Bioscience*

**DOI:** 10.1186/2045-3701-1-1

**Published:** 2011-01-18

**Authors:** Yun-Bo Shi

**Affiliations:** 1 The National Institutes of Health, Bethesda, MD, USA

## Abstract

*Cell & Bioscience *welcomes the submission of your best work for rapid open access publication. This is the official journal of the Society of Chinese Bioscientists in America (SCBA).

## Editorial

Since its founding twenty-six years ago, the Society of Chinese Bioscientists in America (SCBA; http://www.scbasociety.org) has grown to be the largest organization of Chinese bioscientists outside of Asia [1]. The SCBA is constituted by some of the best and brightest bioscientists primarily located in North America and Asia who are prolific in publishing outstanding works in competitive journals. After much deliberation and recognizing the increasing popularity of online open access publishing, the society has determined that now is the right time to launch *Cell & Bioscience*.

*Cell & Bioscience *aims to publish the best works of broad general interest. We have an impeccable collection of dedicated editors and Editorial Board members who will guarantee you fast and fair peer review. We want to publish your articles, and we welcome your submission. Please remember that while *Cell & Bioscience *is the official journal of the SCBA, you do not need to be a society member to submit to and publish in *Cell & Bioscience*.

What distinguishes *Cell & Bioscience *from others? In 2011, one appreciates that perhaps the fastest growing region for scientific research is Asia. This is where a new group of scientists and readers are poised to emerge in large numbers and come online. All journals will strive to target the new demographics, but *Cell & Bioscience *with our roots firmly based in both the United States and China will offer the premier platform for your work to reach both the Western and Asian audience. No other journal can provide you the best of both worlds like *Cell & Bioscience*. Give us a try and see if you don't become a believer!

I want to close with some personal reflections. I am extremely honored to be the founding Editor-in-chief and to lead our outstanding Editorial Board. This journal is the product of long and patient planning by many whose hard work has led finally to its fruition. I would like to thank Chuxia Deng and CC Wang as well as the other journal planning committee members for charting the path for the journal and for their trust in me, Kuan-Teh Jeang for his tireless effort and leadership throughout this process, and the BioMed Central team, especially Elizabeth Bal and Ciaran O'Neill, for their excellent support for the successful, timely launch of the journal. I, the editors, and the *Cell & Bioscience *Editorial Board pledge to work tirelessly to achieve a high visibility journal with excellent impact. But we cannot succeed without your support. We look forward to receiving your manuscript submissions and your advice about *Cell & Bioscience*. I offer you my best wishes for a joyous and successful 2011. Let this be a good year for you and a great year for *Cell & Bioscience*.
